# Improving validity of the trail making test with alphabet support

**DOI:** 10.3389/fpsyg.2023.1227578

**Published:** 2023-07-27

**Authors:** Therese Händel Waggestad, Bjørn Eivind Kirsebom, Carsten Strobel, Anders Wallin, Marie Eckerström, Tormod Fladby, Jens Egeland

**Affiliations:** ^1^Vestfold Hospital Trust, Tønsberg, Norway; ^2^Department of Neurology, University Hospital of North Norway, Tromsø, Norway; ^3^Department of Psychology, The Arctic University of Norway, Tromsø, Norway; ^4^Medical Department, Section of Geriatrics, Memory Clinic and Stroke Unit, Lovisenberg Diaconal Hospital, Oslo, Norway; ^5^Department of Psychiatry and Neurochemistry, Institute of Neuroscience and Physiology, Sahlgrenska Academy, University of Gothenburg, Gothenburg, Sweden; ^6^Department of Psychology, University of Gothenburg, Gothenburg, Sweden; ^7^Department of Neurology, Akershus University Hospital, Lørenskog, Norway; ^8^Institute of Clinical Medicine, University of Oslo, Oslo, Norway; ^9^Department of Psychology, University of Oslo, Oslo, Norway

**Keywords:** trail making test, test-validity, alphabet support, dyslexia, cultural fairness, TMT ratio score, fitness to drive

## Abstract

**Objective:**

The Trail Making Test (TMT) is commonly used worldwide to evaluate cognitive decline and car driving ability. However, it has received critique for its dependence on the Latin alphabet and thus, the risk of misclassifying some participants. Alphabet support potentially increases test validity by avoiding misclassification of executive dysfunction in participants with dyslexia and those with insufficient automatization of the Latin alphabet. However, Alphabet support might render the test less sensitive to set-shifting, thus compromising the validity of the test. This study compares two versions of the TMT: with and without alphabet support.

**Methods:**

We compared the TMT-A, TMT-B, and TMT-B:A ratios in two independent normative samples with (*n* = 220) and without (*n* = 64) alphabet support using multiple regression analysis adjusted for age and education. The sample comprised Scandinavians aged 70–84 years. Alphabet support was included by adding the Latin alphabet A–L on top of the page on the TMT-B. We hypothesized that alphabet support would not change the TMT-B:A ratio.

**Results:**

After adjusting for age and years of education, there were no significant differences between the two samples in the TMT-A, TMT-B, or the ratio score (TMT-B:A).

**Conclusion:**

Our results suggest that the inclusion of alphabet support does not alter TMT’s ability to measure set-shifting in a sample of older Scandinavian adults.

## 1. Introduction

The Trail Making Test (TMT) is one of the more commonly used neuropsychological tests worldwide (Strauss et al., 2006). It measures visual search, processing speed, and executive functions (Tombaugh, 2004) such as set-shifting (Misdraji and Gass, 2010; Salthouse, 2011). The TMT is extensively used as a screening tool for cognitive impairment (Mitrushina et al., 2005) and for assessing driving ability. In particular, TMT-B is regarded a relevant neuropsychological measure for evaluating fitness to drive (Egeto et al., 2019; Holowaychuk et al., 2020). The widespread use of the TMT, together with its central and critical areas of use, makes it imperative to improve its validity.

The TMT has been criticized for misclassifying increased time use by persons with dyslexia or those who have not automatized the Latin alphabet (Avila et al., 2019), as an executive impairment. Persons who learned to read and write using non-Latin alphabets may have learned the Latin alphabet upon immigration to a country that uses this alphabet. However, they may not have automatized it sufficiently and thus may be at a disadvantage when tested on set-shifting. The original TMT consists of Parts A and B. Increased time use when progressing from Part A, consisting only of numbers, to the set-shifting demands of Part B, consisting of both numbers and letters, can be misinterpreted as an executive function (EF) impairment, and not just a lack of automatization of the Latin alphabet. One way to compensate for this is to supply alphabet support in TMT-B.

The TMT first appeared in the Army Individual Test Battery (Partington and Leiter, 1949), and was later included in the Halsted-Reitan Battery (Reitan and Wolfson, 1985). A modified version of the TMT was created and included in the Delis-Kaplan Executive Function System (D-KEFS: Delis et al., 2001). This version included a letter sequencing condition in addition to the number sequencing condition. In the present study, we compare the original TMT from Halsted-Reitan Battery with a revised version of the original TMT Norwegian Revision-3 (TMT-NR3; Strobel et al., 2018). The revision includes alphabet support for TMT-B, where the required part of the Latin alphabet (A–L) is added to the top of the test sheet. However, there are concerns that the inclusion of the alphabet might contaminate the test as a set-shifting test and thus reduce the cognitive complexity of the test.

To investigate whether alphabet support changed the performance among risk groups or if it contaminated the test for healthy adults, Egeland and Follesø (2020) compared TMT-NR3 with the D-KEFS version with no alphabet support. They conducted tests on a Norwegian clinical sample that included a group with “suspected dyslexia.” They created an index of the discrepancy between the number and letter versions of the D-KEFS TMT, and this index predicted a large amount of variance in the letter number version of the D-KEFS, but almost none of the variance in the TMT with letter support, that is, TMT-NR3. This implies that the TMT-NR3 is not as sensitive to difficulties with the alphabet as the D-KEFS version of the TMT. We still need to compare the revised version with the original TMT to investigate whether including alphabet support reduces the test’s cognitive complexity to merely a speeded visual tracking test.

People with dyslexia have been found to spend significantly more time completing both TMT-A and TMT-B (Lima et al., 2011). Rike et al. (2019) investigated whether dyslexia and/or the need for adapted education influenced test performance in TMT-NR3. They found that neither dyslexia nor the need for adapted education were significantly related to TMT-NR3 performance. They also suggested that alphabet support in the revised version made the test more feasible than tests without alphabetic aid.

In the present study, we compared two groups of older adults aged between 70 and 84 years. The first group completed the TMT-NR3 with alphabet support, and the second group completed the original TMT from the Halstead-Reitan battery (Reitan and Wolfson, 1985). The measures in this study were the TMT-A score, TMT-B score, and ratio score (TMT-B:A). The TMT-B:A ratio score reflects the increased complexity of the test when progressing from the first task (TMT-A) of drawing lines between successive numbers to the second task (TMT-B) of drawing lines interchangeably from numbers to letters. Notably, “the task impurity problem” refers to the problem that neuropsychological tests are not function pure. This problem is most evident in testing executive functions that per definition is related to regulating other processes, such as visual search in the case of TMT. When the outcome measure is time spend on solving the task, basal psychomotor speed also influence the score (Burgess, 1997). By controlling for basal speed in visual search, the ratio score synthesizes the net effect of the cost of shifting, and thus minimize the “task impurity” of TMT (Salthouse et al., 2003; Etnier and Chang, 2009) and is considered a good indicator of set-shifting (Lamberty et al., 1994; Arbuthnott and Frank, 2000). The ratio score is also found to minimize the impact of demographic variables such as age and education on performance (Christidi et al., 2015). Ratios around 2.5 are usually found to indicate normal performance (Siciliano et al., 2019) and ratios of more than 3.0 indicate set-shifting impairment (Arbuthnott and Frank, 2000). Therefore, we expect the ratio score to be a good measure of set-shifting, and also expect the ratio score in this study to be close to 2.5.

If listing the alphabet on the top of the page reduces the set-shifting quality of the TMT-B and the test merely focuses on testing speed and visuospatial processing, this is expected to reduce the ratio score. Thus, in the present study, we compared the ratio scores for the two tests hypothesizing that alphabet support will not change the ratio score.

## 2. Materials and methods

### 2.1. Participants

This study included 284 healthy older adults aged 70–84 years old. Informed consent was obtained from all participants. The participants were divided into two groups based on the version of the TMT they completed. The first group (*n* = 220) completed TMT-NR3 and were from the Norwegian Normative Study of Phonemic and Semantic Fluency and Trail Making Test (NorFAST). The second group (*n* = 64) completed the TMT without alphabet support; these participants were recruited from two different studies: 40 from the Dementia Disease Initiation (DDI) and 24 from the Gothenburg MCI study (G-MCI). The DDI and G-MCI were previously combined in a recent normative TMT study (Espenes et al., 2020), and TMT performance did not differ between cohorts.

The recruitment for NorFAST started in 2018 and included individuals from urban and rural areas in all regions of Norway. The inclusion criteria were as follows: participants must be home-dwelling, above 70 years old, not have any known neurological or motor function disorders (self-reported), and have no visual impairment that cannot be corrected with glasses or lenses. They confirmed that they did not have any cognitive deficit beyond what is considered normal aging and that they did not lose their driver’s license for medical reasons. Inclusion of older adults were challenging and therefore, to include more participants over the age of 80, we entered into a cooperation with the municipality of Sandefjord. All home-dwelling persons above 80 years of age were asked to participate in connection with planned home visits to all older adults from the municipality’s health services, thus securing representativeness of the oldest cohort. For this comparative study, we excluded all participants above the age of 84 years from the NorFAST study, as this was the maximum age in the sample from DDI/G-MCI, resulting in a sample size of 225. Moreover, three participants withdrew from the study, and one participant had a history of epileptic seizures and was therefore excluded. The TMT-NR3 manual sets the time limits to 180 s for the TMT-A and 360 s for the TMT-B. No participant used more than 180 s on the TMT-A, but one of the participants in the NorFAST sample used more than 360 s on the TMT-B (396 s) and thus was excluded; finally, 220 participants were included in the NorFAST study.

Participants from the DDI were recruited between January 2013 and October 2018, and the criteria for inclusion in the DDI study (Fladby et al., 2017; Espenes et al., 2020) were as follows: participants must be of ages 40–80 years (although some older adults above 80 years were also included) and be a native speaker of Norwegian, Danish, or Swedish. The exclusion criteria were a history of stroke, severe psychiatric disorder, intellectual disability or developmental disorders, and severe somatic disorders that may influence cognitive functions or subjective symptoms of cognitive decline. Participants were recruited from all Norwegian health regions, primarily through memory clinics and secondarily through responses to advertisements in local media. All participants followed a standardized procedure for assessment, which included standardized neurological and physical examinations, brief neuropsychological assessments, and medical history from the participants and informants. Participants were primarily recruited through spouses and secondarily recruited through self-referrals (Fladby et al., 2017).

The G-MCI participants were recruited between January 2001 and March 2014. The G-MCI study recruited healthy controls mainly through senior citizen organizations; a small proportion were relatives of patients. The inclusion criteria for healthy controls were similar to that of the DDI study; however, the age range was 50–79 years. Individuals with severe somatic diseases and psychiatric disorders that could potentially influence cognitive performance were excluded. All participants aged <70 years in the DDI and G-MCI samples were excluded because this was the lowest age in the NorFAST sample.

### 2.2. Materials

All participants were assessed using TMT-A and TMT-B. In TMT-A, the test-taker should draw a coherent line as fast as possible between successive numbers from 1 to 25 on a sheet of paper. In TMT-B, participants were asked to draw a line interchangeably between numbers 1–13 and letters A–L. In the version with alphabet support, the relevant part of the alphabet (A–L) was printed with 4 mm tall letters on top of the test sheet. We conducted the TMT in the NorFAST-study according to the standardized stimulus material TMT-NR3 (Strobel et al., 2018); meanwhile, the TMT in the DDI/G-MCI study was administered according to the standardized instructions described by Strauss et al. (2006). TMT-NR3 was administered similarly to the original TMT, except that the maximum time for completion of TMT-B was set to 360 s in TMT-NR3 versus 300 s in the original. In the DDI/G-MCI sample, no participants attained the maximum time nor were reported to have aborted the assignment. The ratio score, TMT-B:A, is calculated by dividing time use for TMT-B on time use for TMT-B. This to investigate the relationship between the performance on the two parts of the TMT and to control for inter-individual differences in basal speed and visual search and thus compute a score of the net set-shifting capacity of the subject.

### 2.3. Statistical methods

All statistical analyses were performed using R version 4.03 (RS Team, 2021). Between-group comparisons were performed using independent t-tests for the continuous variables of age and years of education. For the t-tests, Cohen’s d effect sizes are reported for the significant results. For the dichotomous variable “sex,” a chi-square test was performed. As these analyses found that both age and years of education differed between the two groups, multiple regression models with age and years of education as covariates were fitted to assess possible between-group differences in TMT-A, TMT-B, and TMT-B:A performance. Due to a departure from normality (skewness), all TMT measures were log-transformed prior to the analyses. For these models, continuous independent and dependent variables were standardized prior to the analyses, and the coefficients were reported as standardized betas (*β*). The corresponding effect sizes were reported as partial R-square (partial *R*^2^). Results were considered statistically significant at *p* < 0.05. We visually assessed the QQ plots and residuals versus the predicted values to ensure that the assumptions of normality and homoscedasticity were not violated. Table 1 presents the demographic information of the two samples.

**Table 1:** Comparison of demographics and TMT performance between NorFAST and DDI/G-MCI sample.

	DDI/G-MCI *n* = 64	NorFAST *n* = 220	*t*/*x*^2^, *d*, (**p**)
Age Mean (SD) [range]	73.09 (2.79) [70–84]	75.61 (3.99) [70–84]	*t* = 4.73, *d* = 0.56 **(<0.001)**
Years of education Mean (SD) [range]	12.38 (3.69) [6–24]	13.50 (3.25) [7–22]	*t* = 2.37, *d* = 0.28 **(0.018)**
Female *n* (%)	39 (60.94)	124 (56.36)	*x*^2^ = 0.26, (0.612)
TMT-A Mean (SD)	41.75 (11.62)	41.78 (16.01)	*t* = −1.38, (0.168)*
TMT-B Mean (SD)	97.39 (27.25)	101.85 (45.30)	*t* = −0.60, (0.550)*
TMT-B:A Mean (SD)	2.45 (0.82)	2.56 (0.97)	*t* = 0.75, (0.454)*

*n*, number of cases; SD, standard deviation; *t*, *t*-test statistic; *x*^2^, chi-square statistic; *d*, Cohen’s d; *t-test statistic for between-group differences derived from the multiple regression models with age and years of education as covariates.

Bold illustrates significant p-values.

## 3. Results

### 3.1. Main results

**Figure 1:**
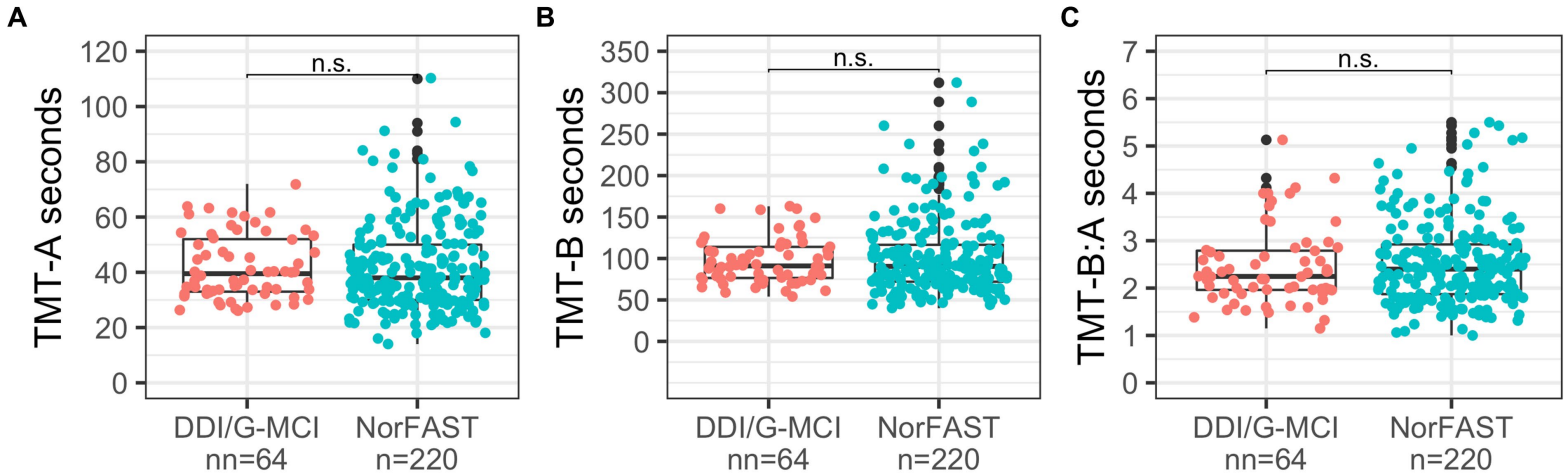
Comparison of TMT-A **(A)**, TMT-B **(B)** and TMT-B:A **(C)** between the DDI/G-MCI and NorFAST samples. Brackets illustrate non-significant (n.s.) group differences in performance at 𝛼=0.05.

Following adjustment for years of education and age, the regression models showed no significant differences between samples for neither TMT-A (*β* = −0.199, *p* = 0.168) and TMT-B performance (*β* = −0.031, *p* = 0.550) nor TMT-B:A ratios (*β* = −0.112, *p* = 0.454). Both ratio scores were close to the expected value of 2.5. Higher educational attainment was associated with faster TMT-A (*β* = −0.147, partial *R^2^* = 0.02, *p* = 0.012) and TMT-B completion times (*β* = −0.255, partial *R^2^* = 0.07, *p* < 0.001) and slightly lower TMT-B:A ratios (*β* = −0.126, partial *R^2^* = 0.02, *p = 0*.037). In contrast, older age was associated with slower completion times for both TMT-A (*β* = 0.249, partial *R^2^* = 0.06, *p* < 0.001) and TMT-B (*β* = 0.26, partial *R^2^* = 0.07, *p* < 0.001) but did not significantly influence the TMT-B:A ratio (*β* = 0.032, *p* = 0.607). Figure 1 illustrates the comparison of the performance of TMT-A, TMT-B, and TMT-B:A ratios in the two samples.

## 4. Discussion

The results showed no significant differences between the two samples tested with versus without alphabet support in terms of performance on TMT-A, TMT-B, or TMT-B:A. This is important because it supports the hypothesis that the two versions of the test should yield the same results for healthy older adults in two independent normative samples.

The ratio score is of particular interest because it measures set-shifting and could be contaminated by offering alphabet support. A lower B:A score on TMT with the alphabet support compared to the original version suggests that the test could be rendered too easy and have a reduced ability to measure set-shifting. However, we found equivalent ratios in both samples at approximately the expected mean ratio of 2.5 (Siciliano et al., 2019) for healthy adults, supporting the hypothesis that including alphabet support does not alter the test’s ability to measure set shifting. Moreover, while age did not significantly influence the ratio score, education did. This is in line with recent normative studies (Siciliano et al., 2019; Espenes et al., 2020) and suggests that as we get older, processing speed declines (Salthouse, 2000), but the decline is slower specifically in executive function (Albinet et al., 2012).

Recruitment to the NorFAST study was conducted based on subjective cognitive status and challenges, and this subjective assessment was our only “measure” of their cognitive level prior to participation. This could lead to the inclusion of participants with cognitive impairment, which could again lead to data contamination. However, recruitment with more comprehensive cognitive testing might lead to the pre-selection of participants with stronger cognitive abilities. Moreover, one participant was excluded from the NorFAST sample based on their TMT performance. The manual was, therefore, considered as a guideline for cut-off with respect to normal performance on the TMT and therefore also functioned as a screening tool for cognitive impairment. Notably, there are very few participants over the age of 80 in the norming samples in general. As a population, we are getting older, and we need far more older adults in our samples. We experienced some challenges in recruiting older participants, and in cooperation with the municipality of Sandefjord, we learned the importance of not making the test situation too extensive for test-takers. The relatively easy and quick implementation of the test was reported as an important reason why participants agreed to participate.

The D-KEFS version of the TMT includes measures of both letter sequencing and number sequencing conditions; therefore, contrasting these measures should give an indication of the participants’ potential challenge with letters, as is the case in dyslexia, or if the participant has not automatized the Latin alphabet. However, as Egeland and Follesø (2020) point out, D-KEFS does not solve the problem with set-shifting, given that difficulty with the Latin alphabet affected the D-KEFS version of TMT-B more than it affected TMT-NR3. Thus, this revision of TMT may offer a solution to the set-shifting challenge.

Several previous studies using the TMT and other neuropsychological tests have concluded that participants with dyslexia are impaired in set-shifting (Moura et al., 2014). In contrast, Doyle et al. (2018) reported difficulties with inhibition and working memory in a group with dyslexia but not with set-shifting. Ferrara et al. (2022) investigated set-shifting in an adolescent group with dyslexia on a task recognized as a “pure” measure of set-shifting. They found that the group with dyslexia had weak-to-moderate, but significantly weakened, performance on this task compared to the control group. They also discussed the variety of findings regarding impaired function related to dyslexia and pointed out that set-shifting is an ability that changes over the course of a lifetime, especially at a young age. They suggested that the large age-gap between participants in different studies of set-shifting in dyslexia resulted in different findings (Ferrara et al., 2022). Smith-Spark et al. (2016) investigated adults with dyslexia because they recognized that most of the research on dyslexia and set-shifting had been conducted on adolescents and children. They found several difficulties with EF such as working memory, inhibition, and set-shifting. Participants also reported subjective difficulties with EF tasks in everyday life. Thus, more research on adults with dyslexia could be valuable but with methods ensuring that the reading impairment characterizing the condition is not confused with EF impairment. Hence, it is important that we, with the intention of correcting for limited knowledge of the Latin alphabet, do not underestimate the genuine impairments that must be considered both in assessing driving ability and for rehabilitation efforts. This must be kept in mind until additional investigations on dyslexia further reveal the different underlying deficits in the diagnosis.

There is an increasing demand for tests to be more culturally fair; in this respect, this revision of the TMT might be valuable. An alternative is the Color Trails Test (CTT: D’Elia et al., 1996) which was developed because of the presumed limited cross-cultural utility of the TMT (Dugbartey et al., 2000). Dugbartey et al. (2000) compared TMT-A and TMT-B with the CTT-1 and CTT-2 for a sample of highly educated non-English speakers. They found significant differences between TMT-B and CTT-2 but not between TMT-A and CTT-1. The authors indicated that the CTT-2 is not an equivalent and more culturally fair version of the TMT, but rather measures different underlying cognitive skills. TMT-NR3 could therefore have value as a cross-cultural measure, but future research should investigate this further.

The purpose of this study was to test whether the TMT with alphabet support changed the set-shifting quality of TMT-B by making it more similar to TMT-A. While this study does not find that to be true, it supports the inclusion of alphabet support in the TMT. This finding suggests that the TMT’s complexity is not lost or reduced. The set-shifting quality of the TMT was the same with or without alphabet support for healthy Norwegian older adults. Alphabet support in the TMT might retain the validity of the measure for people with dyslexia and it might increase cultural fairness for people with insufficient automatization of the alphabet (Egeland and Follesø, 2020), thereby improving the validity and clinical utility of the TMT. Furthermore, alphabet support will also contribute to minimizing the “task impurity” of TMT-B for these participants. The additional time used to remember the alphabet will be reduced and therefore make the TMT-B a more “pure” measure of set-shifting.

The latter argument may also be important for clinical studies. The TMT is often used to stage and monitor patients in clinical trials; for example, Hajjar et al. (2020) used the TMT as the primary outcome for executive function in a randomized clinical trial. Therefore, including patients of different ethnic backgrounds in clinical studies can present a challenge if the Latin alphabet has not been automatized, and excluding these patients may pose ethical issues. Minimizing the “task impurity” and making the TMT more culturally fair can enhance its relevance and validity in clinical studies.

The main limitation of this study is that we compared data across different projects, necessitating controlling for educational differences in the analyses. However, apart from the inclusion of alphabet support, the two versions of the TMT are identical. Moreover, the administration of TMT was similar in both groups. One potential limitation is that the sample sizes differed between the two groups. Nevertheless, the smaller sample size secured sufficient statistical power.

Another limitation is that the inclusion criteria differed between the two groups. The first group relied, to a large degree, on self-reporting of cognitive functioning, and the second group, those who completed the TMT without alphabet support, were tested with the TMT as part of a broader neurocognitive battery. The two groups may have different levels of cognitive function. Therefore, we chose the same age range for the two groups, and we expected any cognitive imbalance between the two groups to be visible in the raw and ratio scores. No differences were observed in the performance of the two samples.

TMT-NR3 is a valuable revision that is easy for clinicians to implement if the participant is considered to be at risk of impaired performance on the TMT because of dyslexia or insufficient automatization of the alphabet. Relevant healthcare professionals can get access to the TMT-NR3 (Strobel et al., 2018) by approaching the Norwegian National Center for aging and health via email on: post@aldringoghelse.no. Notably, use of the revised version is not restricted to these two groups. This study aimed to investigate whether the inclusion of alphabet support compromised the complexity of the TMT. In our study, it did not compromise complexity for the older healthy Scandinavian adults. This indicates that TMT-NR3 can be implemented for all healthy older Scandinavian adults. Future research should also investigate whether this is the case in other age groups. Finally, the Norwegian revision of the TMT is also valuable outside Norway, as TMT-NR3 is based on the Latin alphabet, which is the most commonly used script worldwide (Vaughan, 2020).

## Data availability statement

The raw data supporting the conclusions of this article will be made available by the authors, without undue reservation.

## Ethics statement

The studies involving human participants were reviewed by our regional committee for medical and healthcare research ethics (REK) and were considered to not be in need of their assessment because the project only uses data from healthy persons who volunteer to participate. We therefore have an approval from the Norwegian Center for research data (NSD). The patients/participants provided their written informed consent to participate in this study.

## Author contributions

JE, CS, BK, and TW contributed to the conception and design of the study. BK performed the statistical analysis and wrote those sections of the manuscript. TW wrote the first draft of the manuscript. All authors contributed in the data-collection process and contributed to the manuscript revision, read, and approved the submitted version.

## Funding

NorFAST was supported by the Vestfold Hospital Trust, Vestfold, Norway. G-MCI by the ALF/LUA research grant in Gothenburg, Sweden (ALFGBG-720661) and DDI was funded by the Norwegian Research Council, JPND/PMI-AD (NRC 311993) and Helse-Nord (HNF1540-20).

## Conflict of interest

BK has served as a consultant for Biogen. ME works as independent reviewer for Medavante-Prophase.

The remaining authors declare that the research was conducted in the absence of any commercial or financial relationships that could be construed as a potential conflict of interest.

## Publisher’s note

All claims expressed in this article are solely those of the authors and do not necessarily represent those of their affiliated organizations, or those of the publisher, the editors and the reviewers. Any product that may be evaluated in this article, or claim that may be made by its manufacturer, is not guaranteed or endorsed by the publisher.

## References

[ref1] Albinet C. T. Boucard G. Bouquet C. A. Audiffren M. (2012). Processing speed and executive functions in cognitive aging: how to disentangle their mutual relationship? Brain Cogn. 79, 1–11. doi: 10.1016/j.bandc.2012.02.001, PMID: 22387275

[ref2] Arbuthnott K. Frank J. (2000). Trail making test, part B as a measure of executive control: validation using a set-switching paradigm. J. Clin. Exp. Neuropsychol. 22, 518–528. doi: 10.1076/1380-3395(200008)22:4;1-0;FT518 10923061

[ref3] Avila J. F. Verney S. P. Kauzor K. Flowers A. Mehradfar M. Razani J. (2019). Normative data for Farsi-speaking Iranians in the United States on measures of executive functioning. Appl. Neuropsychol. Adult 26, 229–235. doi: 10.1080/23279095.2017.1392963, PMID: 29313722 PMC8379020

[ref4] Burgess P. (1997). “Theory and methodology in executive function research. Burgess, P.W. (1997) theory and methodology in executive function research” in Theory and methodology of frontal and executive function. ed. Rabbitt P. (East Sussex, UK: Psychology Press), 81–116.

[ref5] Christidi F. Kararizou E. Triantafyllou N. Anagnostouli M. Zalonis I. (2015). Derived Trail making test indices: demographics and cognitive background variables across the adult life span. Aging Neuropsychol. Cognit. 22, 667–678. doi: 10.1080/13825585.2015.1027650 25798536

[ref6] D’elia L. Satz P. Uchiyama C. White T. (1996). Color trails test. Professional manual. Odessa, Fl: Psychological Assessment Resources.

[ref7] Delis D. C. Kaplan E. Kramer J. H. (2001). D‐KEFS Executive Function System: Examiners manual, San Antonio, TX: Psychological Corporation.

[ref8] Doyle C. Smeaton A. F. Roche R. A. Boran L. (2018). Inhibition and updating, but not switching, predict developmental dyslexia and individual variation in Reading ability. Front. Psychol. 9:795. doi: 10.3389/fpsyg.2018.00795, PMID: 29892245 PMC5985558

[ref9] Dugbartey A. T. Townes B. D. Mahurin R. K. (2000). Equivalence of the color trails test and trail making test in nonnative English-speakers. Arch. Clin. Neuropsychol. 15, 425–431. doi: 10.1093/arclin/15.5.425, PMID: 14590218

[ref10] Egeland J. Follesø K. (2020). Offering alphabet support in the trail making test: increasing validity for participants with insufficient automatization of the alphabet. Appl. Neuropsychol. Adult, 29, 478–485. doi: 10.1080/23279095.2020.1774377 32546072

[ref11] Egeto P. Badovinac S. D. Hutchison M. G. Ornstein T. J. Schweizer T. A. (2019). A systematic review and meta-analysis on the association between driving ability and neuropsychological test performances after moderate to severe traumatic brain injury. J. Int. Neuropsychol. Soc. 25, 868–877. doi: 10.1017/S1355617719000456 31084639

[ref12] Espenes J. Hessen E. Eliassen I. V. Waterloo K. Eckerström M. Sando S. B.. (2020). Demographically adjusted trail making test norms in a Scandinavian sample from 41 to 84 years. Clin. Neuropsychol. 34, 110–126. doi: 10.1080/13854046.2020.1829068, PMID: 33034252

[ref13] Etnier J. L. Chang Y.-K. (2009). The effect of physical activity on executive function: a brief commentary on definitions, measurement issues, and the current state of the literature. J. Sport Exerc. Psychol. 31, 469–483. doi: 10.1123/jsep.31.4.469, PMID: 19842543

[ref14] Ferrara M. Benassi E. Camia M. Scorza M. (2022). Application to adolescents of a pure set-shifting measure for adults: identification of poor shifting skills in the group with developmental dyslexia. Mediterr. J. Clin. Psychol. 10, 1–25. doi: 10.13129/2282-1619/mjcp-3406

[ref15] Fladby T. Palhaugen L. Selnes P. Waterloo K. Brathen G. Hessen E.. (2017). Detecting at-risk Alzheimer's disease cases. J. Alzheimers Dis. 60, 97–105. doi: 10.3233/JAD-170231, PMID: 28826181 PMC5611830

[ref16] Hajjar I. Okafor M. Mcdaniel D. Obideen M. Dee E. Shokouhi M.. (2020). Effects of candesartan vs Lisinopril on neurocognitive function in older adults with executive mild cognitive impairment: a randomized clinical trial. JAMA Netw. Open 3, –E2012252. doi: 10.1001/jamanetworkopen.2020.12252, PMID: 32761160 PMC7411539

[ref17] Holowaychuk A. Parrott Y. Leung A. W. (2020). Exploring the predictive ability of the motor-free visual perception test (Mvpt) and trail making test (Tmt) for on-road driving performance. Am. J. Occup. Ther. 74, 7405205070p1–7405205070p8. doi: 10.5014/ajot.119.040626, PMID: 32804625

[ref18] Lamberty G. J. Putnam S. H. Chatel D. M. Bieliauskas L. A. (1994). Derived Trail making test indices: a preliminary report. Neuropsychiatry Neuropsychol. Behav. Neurol. 7, 230–234.

[ref19] Lima R. F. D. Azoni C. A. S. Ciasca S. M. (2011). Attentional performance and executive functions in children with learning difficulties. Psicologia 24, 685–691.

[ref20] Misdraji E. L. Gass C. S. (2010). The trail making test and its neurobehavioral components. J. Clin. Exp. Neuropsychol. 32, 159–163. doi: 10.1080/13803390902881942 19459077

[ref21] Mitrushina M. Boone K. B. Razani J. D'elia L. F. (2005). Handbook of normative data for neuropsychological assessment. New York, NY: Oxford University Press.

[ref22] Moura O. Simões M. R. Pereira M. (2014). Executive functioning in children with developmental dyslexia. Clin. Neuropsychol. 28, 20–41. doi: 10.1080/13854046.2014.964326 25277716

[ref23] Partington J. E. Leiter R. G. (1949). Partington's pathways test. Psychol. Serv. Cen. J. 1, 11–20.

[ref24] Reitan R. Wolfson D. (1985). Neuropsychological test battery: theory and clinical interpretation, Neuropsychology Press, Tuscon, AZ.

[ref25] Rike P.-O. Geirsdottir S. S. Omreng C. Schøyen A.-H. S. Brandt R. Holthe I. L. (2019). Norwegian adolescent reference data for a cognitive screening battery with relevance to driving capacity. J. Norw. Neuropsychol. Soc. 21, 4–12.

[ref33] RS Team, (2021). Rstudio: integrated development environment for R. Boston, MA, Rstudio, PBC.

[ref26] Salthouse T. A. (2000). Aging and measures of processing speed. Biol. Psychol. 54, 35–54. doi: 10.1016/S0301-0511(00)00052-1 11035219

[ref27] Salthouse T. A. (2011). What cognitive abilities are involved in trail-making performance? Intelligence 39, 222–232. doi: 10.1016/j.intell.2011.03.001, PMID: 21789028 PMC3141679

[ref28] Salthouse T. A. Atkinson T. M. Berish D. E. (2003). Executive functioning as a potential mediator of age-related cognitive decline in Normal adults. J. Exp. Psychol. Gen. 132, 566–594. doi: 10.1037/0096-3445.132.4.566, PMID: 14640849

[ref29] Siciliano M. Chiorri C. Battini V. Sant’elia V. Altieri M. Trojano L.. (2019). Regression-based normative data and equivalent scores for trail making test (Tmt): an updated Italian normative study. Neurol. Sci. 40, 469–477. doi: 10.1007/s10072-018-3673-y, PMID: 30535956

[ref30] Smith-Spark J. H. Henry L. A. Messer D. J. Edvardsdottir E. Zięcik A. P. (2016). Executive functions in adults with developmental dyslexia. Res. Dev. Disabil. 53, 323–341. 26970859 10.1016/j.ridd.2016.03.001

[ref31] Strauss E. Sherman E. M. Spreen O. (2006). A compendium of neuropsychological tests: administration, norms, and commentary, New York, Oxford University Press.

[ref32] Strobel C. Johansen H. Aga O. Bekkhus-Wetterberg P. Brierly M. Egeland J.., (2018). Manual Norsk Revidert trail making test (Tmt-Nr3) (Norwegian). Oslo, Norway. Available at: https://www.aldringoghelse.no/skalaer-og-tester/

[ref34] Tombaugh T. N. (2004). Trail making test a and B: normative data stratified by age and education. Arch. Clin. Neuropsychol. 19, 203–214. doi: 10.1016/S0887-6177(03)00039-8, PMID: 15010086

[ref35] Vaughan D. (2020). The World’s 5 Most commonly used writing systems Encyclopedia Britannica. Available at: https://www.britannica.com/list/the-worlds-5-most-commonly-used-writing-systems

